# Sarcopenic Obesity and Activities of Daily Living in Stroke Rehabilitation Patients: A Cross-Sectional Study

**DOI:** 10.3390/healthcare8030255

**Published:** 2020-08-06

**Authors:** Tatsuya Matsushita, Shinta Nishioka, Shiori Taguchi, Anna Yamanouchi, Ryusei Nakashima, Hidetaka Wakabayashi

**Affiliations:** 1 Department of Clinical Services, Nagasaki Rehabilitation Hospital, Nagasaki 850-0854, Japan; wkymf348@yahoo.co.jp (T.M.); ksharu1216@gmail.com (S.T.); s.y.k.o@outlook.jp (A.Y.); r-nakashima@zeshinkai.or.jp (R.N.); 2 Department of Clinical Nutrition and Food Services, Nagasaki Rehabilitation Hospital, Nagasaki 850-0854, Japan; 3 Department of Rehabilitation Medicine, Tokyo Women’s Medical University Hospital, Tokyo 162-8666, Japan; noventurenoglory@gmail.com

**Keywords:** activities of daily living, rehabilitation, sarcopenia, sarcopenic obesity, stroke

## Abstract

Reports investigating the relationship between sarcopenic obesity and activities of daily living in older patients with stroke were limited. This study aimed to examine the prevalence of sarcopenic obesity and its association with activities of daily living in older post-stroke patients in convalescent rehabilitation wards. This study was performed in older post-stroke patients admitted to convalescent rehabilitation wards between January 2017 and March 2019. Sarcopenia was diagnosed based on the skeletal muscle mass index and hand grip strength according to the criteria of the 2019 Asian Working Group for Sarcopenia. Obesity was diagnosed according to the body fat percentage; ≥27% in men, ≥38% in women. The primary outcome was the Functional Independence Measure (FIM) score upon admission, which was analyzed using multiple linear regression. In total, 376 participants (women 44%; mean age 77.5 years) were analyzed and classified as normal (22%), simple obesity (17%), sarcopenia without obesity (32%), and sarcopenic obesity (28%). The presence of sarcopenic obesity was independently associated with the FIM score (95% CI, −16.157 to −5.353), whereas simple obesity and sarcopenia without obesity were not. In conclusion, sarcopenic obesity was independently associated with lower activities of daily living capability in older patients with stroke.

## 1. Introduction

Obesity is frequently observed in patients with stroke and results in adverse outcomes. Remarkably, 19.4–31.4% of patients after stroke are classified as obese, and this varies with setting [1,2,3]. Obesity is a known risk factor for stroke and is closely associated with hypertension and diabetes, leading to increased risk of stroke onset [4]. Loss of muscle mass commonly occurs after stroke and is accompanied by increased fat mass deposition in the paretic limb [5,6,7]. Notably, decreased muscle mass and strength and increased fat mass are also observed in the nonparetic limb during a similar time course after stroke [8,9]. Therefore, fat mass in the paretic limb and whole body may increase after stroke, resulting in increased risk of recurrent stroke [10].

However, the effect of overweight and obesity on the onset of cerebrovascular disease is controversial as overweight and obesity are associated with better survival after stroke (obesity paradox) [11,12]. In addition, obesity has been previously reported to be independently correlated with the functional recovery of patients with stroke in the convalescent ward [13]. Although the causal relationship between excessive body weight and better functional recovery remains unclear, this phenomenon can be explained by coexisting muscle mass and function as obese patients often have higher muscle mass than those with normal weight or underweight. In contrast, obese patients with low muscle mass or muscle strength, namely, sarcopenic obesity (SO), showed poor outcomes including in activities of daily living (ADL) [14,15,16,17]. In addition, sarcopenia more likely develops among stroke patients, because approximately half of patients with stroke had sarcopenia, (i.e., stroke-related sarcopenia) in convalescent rehabilitation wards, which may be associated with good rehabilitation outcome [5,18,19]. Therefore, obese patients with sarcopenia may have poorer prognosis than those without.

Previously, stroke patients with sarcopenia showed poor improvement of ADL capacity, particularly in males [18]. However, as the previous study did not focus on obesity and did not examine the indicators of fat mass, the prevalence of SO and its relationship with the ability to perform ADL remains unclear. Because SO is strongly associated with decreased ADL in community-dwelling older adults, we hypothesized that SO also has more of a negative impact on ADL in post-stroke patients than does simple obesity.

Therefore, a cross-sectional study was conducted to clarify the relationship among SO, simple obesity, and ADL capability in patients with stroke in convalescent rehabilitation wards.

## 2. Materials and Methods

### 2.1. Study Participants

A cross-sectional study was performed at convalescent rehabilitation wards in a single center in Japan. A total of 498 consecutive patients aged ≥65 years admitted for post-stroke rehabilitation between January 2017 and March 2019 were included. Patients with disturbed consciousness, metallic implantation, or missing data were excluded. All data were extracted from the medical chart.

Convalescent rehabilitation wards aim to maximize the recovery of ADL capability and enable patients to return to their own homes by utilizing the skills of multidisciplinary rehabilitation teams that involve medical doctors, nurses, physical therapists, occupational therapists, speech–language–hearing therapists, social workers, registered dietitians, care workers, dental hygienists, and pharmacists [20]. Healthcare cost of the wards was covered by the public healthcare insurance for up to 180 days.

### 2.2. Data Collection

Basic information was recorded for each of the study participants upon admission, including age, sex, stroke subtype (cerebral infarction, intracerebral hemorrhage, and subarachnoid hemorrhage), days from stroke onset to admission in convalescent rehabilitation wards, certification of public long-term care insurance before the stroke onset, lower limb motor paralysis, body mass index (BMI), and Mini Nutritional Assessment^®^–Short Form (MNA^®^–SF) [21,22]. The MNA^®^–SF was evaluated on the day of admission by a registered dietitian. Patients were classified into three groups based on the MNA–SF score. Original cutoff values are 0–7 (malnourished), 8–11 (at risk of malnutrition), and 12–14 (well-nourished) [22]. However, the probability of a false-positive case may increase if the cutoff values are applied for patients in the rehabilitation setting [23]. Therefore, modified cutoff values for stroke patients undergoing rehabilitation were used: 0–5 (malnourished), 6–7 (at risk of malnutrition), and 8–14 (well-nourished) in addition to the original ones because it showed sufficient validity [24]. Certification for public long-term care insurance was classified into five categories based on national public policy, from “Care level 1,” can perform almost all ADL, but required partial care, to “Care level 5,” must be aided in all situations and had difficulty with communication. Degree of lower limb motor paralysis was evaluated using the Brunnstrom recovery stage system (BRS) by physical therapists [25]. Patients were classified into three groups: BRS I to IV, BRS V to VI, and “absence” [26]. Within three days of admission, hand grip strength (HG), bioelectrical impedance analysis (BIA) for skeletal muscle and fat mass, and the Functional Independence Measure (FIM) scores for physical and cognitive functions were measured [27]. HG was measured using the Smedley-type digital hand–dynamometer grip–D (Takei Scientific Instruments Co., Ltd., Niigata, Japan) in the dominant hand (or in the case of hemiparesis, in the non-paralyzed hand) by physical therapists. Patients were required to be seated during measurement, depending on their ability, and with arms straight at their side; the higher value from two measurements was recorded. BIA was evaluated using the InBody S10 (InBody, Tokyo, Japan) in the supine position by physical therapists or registered dietitians. Patients with pacemaker implants were not measured by BIA.

### 2.3. Main Outcomes

The primary outcome was the FIM score, comprising 13 subitems of motor domain (FIM–motor) and five subitems of the cognitive domain (FIM–cognitive) [27]. Each element is assigned with 1 to 7 points, where 1 indicates total assistance and 7 indicates complete independence. The total FIM score ranges from 18 to 126 points accordingly. Physical, occupational, speech–language–hearing therapists, nurses, and care workers assessed the FIM score. Secondary outcomes were FIM–motor and FIM–cognitive.

### 2.4. Definition of Sarcopenic Obesity

Since a globally accepted SO definition has not been established [28], patients fulfilling the criteria for both sarcopenia and obesity were identified as being SO in this study. Sarcopenia was diagnosed when patients had both a low skeletal muscle mass index (SMI) measured by BIA and low muscle strength measured by HG, based on the Asian Working Group for Sarcopenia (AWGS) [29]. Furthermore, elderly patients with stroke often have difficulty walking; therefore, physical performance tests such as the 6 m walking test were not used to define sarcopenia in this study. SMI was calculated from the estimated appendicular muscle mass (kg) divided by the squared height (m). The cutoff values for HG to define sarcopenia were <28 kg and <18 kg in men and women, and those of SMI were <7.0 kg/m^2^ and <5.7 kg/m^2^ in men and women, respectively, based on the AWGS2019 criteria [29]. The cutoff values for body fat percentage to define obesity were ≥27 % and ≥38 % in men and women, respectively, based on a previous study [14].

### 2.5. Sample Size Calculation

A study size analysis was performed using the Power and Sample Size Calculation software version 3.0 (William D Dupont PhD and Walton D Plummer, Department of Biostatistics, Vanderbilt University School of Medicine, Nashville, TN, USA). A previous Japanese study identified the mean FIM of patients with stroke in convalescent rehabilitation wards to be 68.2 ± 31.2 [20]. Although available data to detect the clinically relevant FIM–total score in convalescent patients with stroke were limited, our previous study [18] demonstrated that the mean difference of the FIM–total score on admission to the convalescent rehabilitation ward between with and without sarcopenia groups was 33.5. Therefore, we inferred that the mean FIM–total score difference was 33.5 between the normal and SO groups. When the ratio is 1:1, 25 patients are needed in each group (normal and SO) for 90% power and alpha error of 0.05. This study requires at least 100 patients to be compared in four groups accordingly: normal, obese, sarcopenia, and SO.

### 2.6. Statistical Analysis

Results are reported as means and standard deviations (SD) for normally distributed variables, medians and interquartile ranges (IQR) for skewed distributed variables, and number (%) for categorical data. Between-group comparisons were performed using analysis of variance, chi-square test, and Kruskal–Wallis test. Post-hoc comparisons were carried out with Tukey’s test, Bonferroni correction, and Dunn’s test. Multiple linear regression analysis was used to determine whether SO, simple obesity, and sarcopenia without obesity were independently associated with FIM, FIM–motor, and FIM–cognitive. From the clinical point of view, age, sex, stroke subtype, days from stroke onset to admission in convalescent rehabilitation wards, certification for public long-term care insurance before the stroke onset, lower limb motor paralysis, and MNA^®^–SF were considered as covariates. The *p* values of <0.05 were considered statistically significant. All statistical analyses were conducted using IBM SPSS version 21 (Armonk, NY, USA).

### 2.7. Ethical Consideration

This study was conducted in accordance with the Declaration of Helsinki and approved by the ethical committee of Nagasaki Rehabilitation Hospital (approval number: H31–02). Because of the anonymous nature of the data, the requirement for informed consent was exempted. We supplied an opt-out option to allow patients to withdraw from the database instead.

## 3. Results

**Figure 1:**
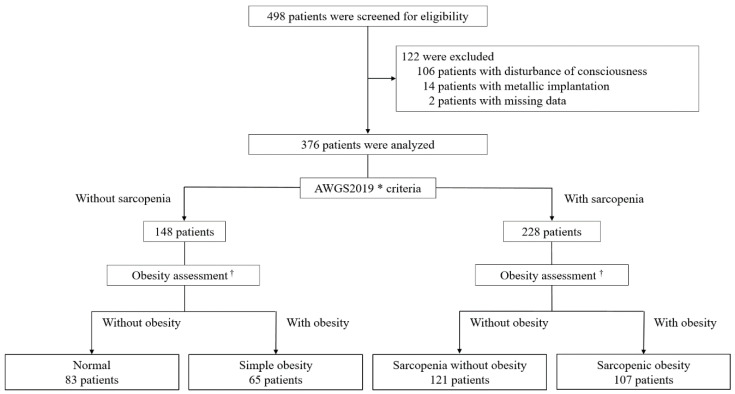
Flowchart of study participants. * AWGS, Asian Working Group for Sarcopenia 2019. ^†^ The cutoff values for body fat percentage to define obesity were ≥27% and ≥38% in men and women, respectively.

Among 498 patients screened for study participation, those with disturbed consciousness (*n* = 106), metallic implantation (*n* = 14), and missing data (*n* = 2) were excluded. Finally, 376 patients were included in the study (mean age: 77.5 years; 210 men and 166 women) (Figure 1). Approximately 72% (272/376) of patients were diagnosed with cerebral infarction, whereas 23% (87/376) of them were diagnosed with intracerebral hemorrhage and 5% (17/376) with subarachnoid hemorrhage.

The study patient characteristics are summarized in Table 1. Among 376 patients, 46% (172/376) were obese (mean age: 77.7 years; 117 men and 55 women) and 28% (107/376) were defined as SO. Based on the modified cutoff values of the MNA^®^–SF, 36% (135/376), 28% (106/376), and 36% (135/376) of patients were malnourished, at risk of malnutrition, and well-nourished, respectively. Post-hoc multiple comparisons showed that patients with SO were significantly older (*p* < 0.001) and had higher BMI (*p* < 0.001) and body fat percentage (*p* = 0.009) compared with those with simple obesity. Additionally, sarcopenic patients without obesity showed a significantly higher proportion of malnutrition than those in other groups (*p* < 0.001).

Table 2 and Table 3 show the multivariate analysis results on the score of FIM–total, FIM–motor, and FIM–cognitive. SO was independently associated with the FIM–total (partial regression coefficient, −10.755; 95% confidence interval (CI), −16.157 to −5.353), as well as age, intracerebral hemorrhage, care needed before the stroke onset, lower limb motor paralysis, and MNA–SF. In contrast, simple obesity and sarcopenia without obesity were not independent explanatory variables for the FIM–total score. The presence of SO was also associated with the FIM–motor score (partial regression coefficient, −7.876; 95% CI, −12.067 to −3.685) and the FIM–cognitive score (partial regression coefficient, −2.879; 95% CI, −4.743 to −1.014).

## 4. Discussion

This cross-sectional study investigated the prevalence of SO among patients with stroke and its association with ADL capability in convalescent rehabilitation wards, and found two important clinical observations. First, approximately 30% of patients with stroke in convalescent rehabilitation wards had SO. Second, SO was independently associated with lower ADL capability in this population.

In our study, 28% of patients with stroke in rehabilitation wards presented SO. To the best of our knowledge, this is the first study to show the prevalence of SO in patients after stroke in a convalescent rehabilitation ward. The prevalence of SO varies among different settings. In a community-dwelling German population aged ≥70 years, it ranged from 2.1–4.1% in men to 0–2.3% in women [30,31]. In a cohort from South Korea, the prevalence of SO in people aged ≥60 years ranged from 1.3–20.3% in men to 0.8–16.5% in women [32]. The US cohort using National Health and Nutrition Examination Survey data also showed that 12.6% of men and 33.5% of women had SO [33]. Our study indicated that patients with stroke in a rehabilitation setting have higher prevalence of SO than do older adults in community-dwellings. However, the definition of SO varied within the reported studies, making the comparison of their prevalence difficult. Conversely, patients with SO were found to be significantly older, tended to be malnourished, had higher BMI and body fat percentage, and were more dependent before the onset than patients with simple obesity. These findings are similar to that of the previous study on patients with stroke [18], where patients with sarcopenia were older, malnourished, and more dependent before the onset than those without sarcopenia.

The presence of SO was independently associated with low ADL capability. These findings are consistent with those of previous studies, showing a significant relationship between SO and decreased ADL among older adults in the community [14,15,16,17]. SO may cause insulin resistance due to intramuscular fat accumulation [34,35]. Hyperinsulinemia has been shown to increase the amount of serum myostatin, which acts to negatively regulate the skeletal muscle growth [36]. Moreover, fast-type muscle fibers switch to slow-type muscle fibers resulting in decreased muscle mass and strength [34,37]. Additionally, sex-specific hormonal changes, inflammatory pathways, and myocellular mechanisms impair the muscle function in older adults with obesity [38]. Conversely, simple obesity and sarcopenia without obesity were not associated with decreased ADL. However, previous studies reported that the presence of sarcopenia in patients with stroke was associated with worse recovery of physical function [18,19]. One reason for this inconsistency is that previous studies did not differentiate SO and sarcopenia without obesity; therefore, their results might not represent the effect of sarcopenia in the non-obese population. Hence, healthcare professionals should assess and treat not only sarcopenia but also SO in patients with stroke in a convalescent rehabilitation ward.

We found that patients classified as having SO and sarcopenia without obesity had higher prevalence of cognitive deterioration. In addition, SO was independently associated with low FIM–cognitive. This result is in accordance with previous studies that indicated SO may possibly present as a risk factor for cognitive deterioration [39,40,41]. Chronic inflammation, oxidative stress, and insulin resistance may contribute to the correlation between SO and cognitive deterioration [42,43,44].

This study had some limitations. First, a causal relationship between SO and ADL capability was unknown due to the current study design. Thus, longitudinal studies are needed to clarify this relationship. Second, the current study did not include a variable for stroke severity, which is closely associated with functional outcomes. Instead, this study included the BRS, which is associated with stroke severity [45].

## 5. Conclusions

In this study, we found that approximately 46% of the stroke patients in convalescent rehabilitation wards were obese and that 28% were defined as SO. Patients with SO were significantly older (*p* < 0.001), had a higher BMI (*p* < 0.001), and had a higher body fat percentage (*p* < 0.001) compared to those with simple obesity. In addition, SO was independently associated with ADL capability in this population: B = −10.755, 95% CI = −16.157 to −5.353 (for FIM–total); B = −7.876, 95% CI = −12.067 to −3.685 (for FIM–motor); B = −2.879, 95% CI = −4.743 to −1.014 (for FIM–cognitive). SO may be more clinically significant than simple obesity among stroke patients in rehabilitation wards. Body composition should be routinely assessed based on the usual rehabilitation assessments in rehabilitation settings. However, further research is needed to investigate the effects of SO on ADL and effective intervention for SO in patients with stroke.

## Figures and Tables

**Table 1:** Characteristics of patients with stroke in convalescent rehabilitation wards and between-group comparison according to the presence of obesity and/or sarcopenia.

Variables	Normal *n* = 83	Simple Obesity *n* = 65	Sarcopenia without Obesity *n* = 121	Sarcopenic Obesity *n* = 107	*p* Value
Age, y, mean (SD)	73.5 (7.2)	74.1 (6.4)	80.0 (8.1) *^,d^	80.0 (7.3) *^,d^	<0.001 ^a^
Sex (men), n (%)	46 (55)	50 (77) ^‡,e^	47 (39) ^†,e^	67 (63)	<0.001 ^b^
Stroke subtype, n (%)					0.17 ^b^
Cerebral infarction	65 (78)	49 (75)	79 (65)	79 (74)	
Intracerebral hemorrhage	13 (16)	16 (25)	35 (29)	23 (22)	
Subarachnoid hemorrhage	5 (6.0)	0 (0)	7 (5.8)	5 (4.7)	
Days from onset, d, median [IQR]	22 (17–34)	23 (19–299)	26 (20–35)	23 (18–31)	0.13 ^c^
Care needed before stroke onset, n (%)	7 (8.4)	5 (7.7)	29 (24) *^,e^	28 (26) *^,e^	0.001 ^b^
Lower limb motor paralysis, n (%)					0.74 ^b^
BRS I–IV	16 (19)	12 (19)	31 (26)	23 (22)	
BRSV–VI	50 (60)	34 (52)	65 (54)	59 (55)	
absence	17 (21)	19 (29)	25 (21)	25 (23)	
Handgrip strength, kg, mean (SD)	24.9 (7.7)	27.4 (7.5)	14.9 (6.2) *^,d^	16.6 (6.0) *^,d^	<0.001 ^a^
Body mass index, kg/m^2^, mean (SD)	21.1 (2.0) ^‖,d^	24.9 (2.8) ^‖,d^	19.1 (2.3) ^‖,d^	23.1 (2.7) ^‖,d^	<0.001 ^a^
Skeletal muscle mass index, kg/m^2^, mean (SD)	6.4 (0.9)	6.8 (0.9)	5.1 (0.9) *^,d^	5.5 (0.8) *^,d^	<0.001 ^a^
Body fat percentage, mean (SD)	25.5 (6.8) ^†,d^	34.9 (5.4)	26.8 (7.0) ^†,d^	38.1 (6.5) ^‖,d^	<0.001 ^a^
MNA^®^–SF, n (%)					<0.001 ^b^
0–5 (Malnourished) ^g^	24 (29)	9 (14) ^†,e^	66 (55) ^‖,e^	36 (34) ^§,e^	
6–7 (At risk of malnutrition) ^g^	21 (25)	17 (26)	35 (29)	33 (31)	
8–14 (Well-nourished) ^g^	38 (46)	39 (60) ^†,^^e^	20 (17) ^‖,^^e^	38 (36) ^§,e^	
8–11 ^h^	38 (46)	35 (54)	20 (17)	38 (36)	
12–14 ^h^	0 (0)	4 (6)	0 (0)	0 (0)	
FIM on admission, score, median [IQR]					
Total	89 (71–107)	92 (74–110.5)	71 (46.5–88.5) *^,f^	71 (46–86) *^,f^	<0.001 ^c^
Motor	60 (46–78)	69 (48–81)	47 (27.5–63) *^,f^	47 (29–60) *^,f^	<0.001 ^c^
Cognitive	27 (21–32)	29 (25–33)	22 (16–28) *^,f^	21 (16–28) *^,f^	<0.001 ^c^

BRS, Brunnstrom recovery stage; MNA^®^–SF, Mini Nutritional Assessment^®^–Short Form; FIM, Functional Independence Measure. ^a^ One-way analysis of variance, ^b^ Chi-square test, ^c^ Kruskal–Wallis test, ^d^ Tukey’s test, ^e^ Bonferroni correction, ^f^ Dunn’s test, ^g^ Based on the modified cut-off values (ref. [23]), ^h^ Based on the original cut-off values (ref. [22]). * *p* < 0.05 for normal and simple obesity groups. ^†^ *p* < 0.05 for simple obesity and sarcopenic obesity groups. ^‡^ *p* < 0.05 for normal and sarcopenia with obesity groups. ^§^ *p* < 0.05 for simple obesity group. ^‖^ *p* < 0.05 for other groups.

**Table 2:** Multivariate linear regression analysis for the FIM–total score *.

Variables	B	95% Confidence Interval	*p* Value
(Constant)	110.311	87.408 to 133.214	<0.001
Simple obesity	1.064	−4.865 to 6.994	0.72
Sarcopenia without obesity	−2.821	−8.265 to 2.623	0.31
Sarcopenic obesity	−10.755	−16.157 to −5.353	<0.001
Age	−0.492	−0.762 to −0.223	<0.001
Women	−0.087	−3.978 to 3.804	0.97
Stroke subtype			
Cerebral infarction (reference)	-	-	-
Intracerebral hemorrhage	−7.286	−11.977 to −2.596	0.002
Subarachnoid hemorrhage	−3.306	−12.573 to 5.961	0.48
Days from onset to admission	−0.122	−0.297 to 0.052	0.17
Care needed before stroke onset	−10.646	−15.575 to −5.717	<0.001
Lower limb motor paralysis			
Absence (reference)	-	-	-
BRS I–IV	−33.240	−38.854 to −27.626	<0.001
BRS V–VI	−9.876	−14.393 to −5.359	<0.001
MNA^®^–SF, points	4.097	3.274 to 4.920	<0.001

FIM, Functional Independence Measure; B, partial regression coefficient; BRS, Brunnstrom recovery stage; MNA^®^–SF, Mini Nutritional Assessment^®^–Short Form. * R^2^ = 0.600.

**Table 3:** Multivariate linear regression analysis for the FIM–motor domain score and FIM–cognitive domain score *.

Variables	FIM–Motor Domain Score *		FIM–Cognitive Domain Score ^†^	
	B	95% Confidence Interval	B	95% Confidence Interval
(Constant)	78.136 ^‡^	60.368 to 95.903	32.176 ^‡^	24.270 to 40.081
Simple obesity	−0.452	−5.052 to 4.148	1.517	−0.530 to 3.563
Sarcopenia without obesity	−2.052	−6.275 to 2.171	−0.769	−2.648 to 1.110
Sarcopenic obesity	−7.876 ^‡^	−12.067 to −3.685	−2.879 ^‡^	−4.743 to −1.014
Age	−0.370 ^‡^	−0.579 to −0.161	−0.122 ^‡^	−0.215 to −0.029
Women	−0.802	−3.820 to 2.217	0.715	−0.628 to 2.058
Stroke subtype				
Cerebral infarction (reference)	-	-	-	-
Intracerebral hemorrhage	−5.686 ^‡^	−9.325 to −2.048	−1.600	−3.219 to 0.019
Subarachnoid hemorrhage	−3.864	−11.053 to 3.324	0.558	−2.640 to 3.757
Days from onset to admission	−0.030	−0.166 to 0.105	−0.092 ^‡^	−0.152 to −0.032
Care needed before stroke onset	−8.020 ^‡^	−11.843 to −4.196	−2.626 ^‡^	−4.328 to −0.925
Lower limb motor paralysis				
Absence (reference)	-	-	-	-
BRS I–IV	−29.330 ^‡^	−33.685 to −24.975	−3.910 ^‡^	−5.848 to −1.972
BRS V–VI	−8.324 ^‡^	−11.829 to −4.820	−1.552	−3.111 to 0.008
MNA^®^–SF, points	3.127 ^‡^	2.489 to 3.765	0.970 ^‡^	0.686 to 1.254

FIM, Functional Independence Measure; B, partial regression coefficient; BRS, Brunnstrom recovery stage; MNA^®^–SF, Mini Nutritional Assessment^®^–Short Form. * R^2^ = 0.613. ^†^ R^2^ = 0.376. ^‡^ *p* < 0.05.
